# Factors affecting malnutrition in children and the uptake of interventions to prevent the condition

**DOI:** 10.1186/s12887-015-0496-3

**Published:** 2015-11-19

**Authors:** Edem M. A. Tette, Eric K. Sifah, Edmund T. Nartey

**Affiliations:** Department of Community Health, School of Public Health, University of Ghana, P.O. Box 4236, Accra, Ghana; Princess Marie Louis Children’s Hospital (PML), P.O. Box GP 122, Accra, Ghana; World Health Organisation Collaborating Centre for Advocacy and Training in Pharmacovigilance, Centre for Tropical Clinical Pharmacology & Therapeutics, School of Medicine and Dentistry, University of Ghana, P. O. Box GP 4236, Accra, Ghana

**Keywords:** Malnutrition, Children, Prevention, Diarrhoea, Risk factors, Interventions

## Abstract

**Background:**

Malnutrition is a major cause of child morbidity and mortality. There are several interventions to prevent the condition but it is unclear how well they are taken up by both malnourished and well nourished children and their mothers and the extent to which this is influenced by socio-economic factors. We examined socio-economic factors, health outcomes and the uptake of interventions to prevent malnutrition by mothers of malnourished and well-nourished in under-fives attending Princess Marie Louise Children's Hospital (PML).

**Methods:**

An unmatched case control study of malnourished and well-nourished children and their mothers was conducted at PML, the largest facility for managing malnutrition in Ghanaian children. Malnourished children with moderate and severe acute malnutrition were recruited and compared with a group of well-nourished children attending the hospital. Weight-for-height was used to classify nutritional status. Record forms and a semi-structured questionnaire were used for data collection, which was analysed with Stata 11.0 software.

**Results:**

In all, 182 malnourished and 189 well-nourished children and their mothers/carers participated in the study. Children aged 6–12 months old formed more than half of the malnourished children. The socio-demographic factors associated with malnutrition in the multivariate analysis were age ≤24 months and a monthly family income of ≤200 GH Cedis. Whereas among the health outcomes, low birth weight, an episode of diarrhoea and the presence of developmental delay were associated with malnutrition. Among the interventions, inadequate antenatal visits, faltering growth and not de-worming one's child were associated with malnutrition in the multivariate analysis. Immunisation and Vitamin A supplementation were not associated with malnutrition. Missed opportunities for intervention were encountered.

**Conclusion:**

Poverty remains an important underlying cause of malnutrition in children attending Princess Marie Louise Children’s Hospital. Specific and targeted interventions are needed to address this and must include efforts to prevent low birthweight and diarrhoea, and reduce health inequalities. Regular antenatal clinic attendance, de-worming of children and growth monitoring should also be encouraged. However, further studies are needed on the timing and use of information on growth faltering to prevent severe forms of malnutrition.

## Background

Malnutrition is regarded as the most important risk factor for illness and death globally and it is associated with 52.5 % of all deaths in young children [1–4]. According to UNICEF, WHO and the World Bank, out of the 161 million under-fives estimated to be stunted globally in 2013, over a third resided in Africa [5]. In addition, about one-third of the 51 million under-fives who were wasted and the 99 million who were underweight were also from Africa [5]. Furthermore, although there has been a global decline in underweight from 25 % to 15 %, Africa has experienced the smallest relative decrease in prevalence going from 23 % in 1990 to 17 % by 2013 [5].

In children, low birth weight, feeding problems, diarrhoea, recurrent illness, measles, pertussis, and chronic disease among others increase the risk of malnutrition [6–8]. These factors vary from locality to locality and children under five years are most at risk. Social factors also have an influence on malnutrition and in the 1990’s, malnutrition was associated with young mothers and low maternal socio-economic status at Princess Marie Louise Children’s Hospital (PML) [6].

The consequences of malnutrition are many and have been extensively documented [2–4, 8, 9]. It includes increased risk of infection, death, and delayed cognitive development, leading to low adult incomes, poor economic growth and intergenerational transmission of poverty [9]. Children with malnutrition have reduced ability to fight infection and are more likely to die from common diseases such as malaria, respiratory infections and diarrhoeal diseases [2–4, 8]. Children who are born with low birth weight and have intrauterine growth retardation, are at increased risk of morbidity and mortality, and other forms of malnutrition compared to healthy infants. They also tend to develop non-communicable diseases such as diabetes and hypertension in adult life [10]. Interventions for reducing malnutrition must therefore begin before birth.

Reproductive Health Services provide the settings for political strategies that can reduce low birth weight by enhancing birth spacing and reducing teenage pregnancy [11–13]. Maternal malnutrition, low gestational weight gain, weight loss due to illness, medical conditions during pregnancy such as malaria, hypertension, smoking, drug and alcohol use, increase the risk of low birth weight [10]. Antenatal care provides the setting to identify and treat such high-risk pregnancies and it offers nutritional and educational interventions which can promote healthy eating habits, hygienic practices and lifestyle changes to reduce low birth weight [10]. Thus low birth weight can be a measure of success in preventing malnutrition during pregnancy through antenatal care.

Promotion of breastfeeding, appropriate complementary feeding, vitamin A supplementation and case management of malnutrition are most effective at preventing malnutrition or its effects [11, 14]. De-worming programmes and conditional cash transfer have been reported to be effective only in specific situational context, while there is little evidence for the effectiveness of interventions such as growth monitoring. Intervention such as immunization and education on clean hygienic practices and nutritional counselling at post-natal and child welfare clinics can also prevent malnutrition [15].

Repeated attacks of diarrhoea and infections leads to weight loss and compromise a child’s nutritional status [1, 15]. This in turn makes the child vulnerable to infections and further weight loss, eventually leading to severe malnutrition unless the cycle is broken. Thus recurrent diarrhoea and sickness episodes reflect the effectiveness of health interventions to prevent and manage diarrhea and infections, and hence prevent malnutrition.

Ghana has several policies and programmes to reduce malnutrition [16, 17]. This includes reproductive health interventions such as antenatal and postnatal care and interventions contained in the Under Fives Child Health Programme. The latter includes promotion of breast feeding, appropriate complementary feeding, growth monitoring, Vitamin A supplementation and immunisation. Others are regular de-worming and strategies for feeding children with special nutritional requirements such as infants of mothers with HIV infection or AIDS [17]. The programme also provides information on appropriate treatment of childhood illnesses such as diarrhoeal diseases [11, 14, 17].

In recent times there has been renewed interest in preventing malnutrition however there is insufficient data on the uptake of these health interventions and the factors which affect them. According to UNICEF the main causes of childhood malnutrition can be categorized into three main underlying factors which are; household food insecurity, inadequate care and unhealthy household environment, and lack of health care services [18]. These in turn are affected by income, poverty, employment, dwelling, assets, remittances, pensions and transfers which are also determined by socio-economic and political factors.

Interventions to prevent malnutrition must target these underlying causes. Thus we examined social factors, health outcomes and the uptake of interventions to prevent malnutrition by mothers of malnourished and well-nourished children under the age of five years attending PML.

## Methods

### Study design

An unmatched case–control study was conducted at the Princess Marie Louise Children’s Hospital in Accra. Cases were defined as children under the age of 5 years with either Moderate Acute Malnutrition (MAM- a weight for height Z score of ≥ −3SD to < − 2 SD) or Severe Acute Malnutrition (SAM-a weight for height Z score of < − 3 SD with or without bilateral pitting oedema). The controls were children under the age of 5 years with well-nourished nutritional status (a weight for height Z scores > − 2SD). The study was part of a larger study which also examined feeding practices, maternal, social, medical and biologic factors associated with malnutrition. We present here the extent of exposure of these children and their mothers to selected health interventions that prevent the malnutrition and the socio-demographic and health outcomes affecting them.

### Study setting

Princess Marie Louise Children’s Hospital is the largest centre dedicated to treating children with malnutrition in the country. The hospital is a 74 bed children’s hospital situated in the commercial centre of the capital, Accra. It provides both primary care and specialized paediatric services for patients brought in by their parents and referrals from health facilities in other parts of Accra and from other regions. In 2012, there were 157 admissions for MAM and SAM at PML with a mortality rate of 11.7 % as reported by the Dietetic unit. The WHO protocol informs case management at the hospital.

### Study population

Patients with malnutrition were identified initially by measuring the Mid Upper Arm Circumference (MUAC) as this is the main measurement used for admitting and identifying patients with SAM and MAM in Ghanaian nutritional rehabilitation centres. Those with Severe Acute malnutrition (SAM), a weight for height Z score of < − 3 SD with or without bilateral pitting oedema (WHO) and Moderate Acute Malnutrition (MAM), a weight for height Z score of ≥ −3SD to < − 2 SD (WHO) were included as cases [19, 20]. Patients with a weight for height Z scores > − 2SD presenting with other conditions were included as controls.

Children who met MUAC criteria but did not meet weight for height criteria or had missing weight or height measurements were excluded from the study. Children with chronic diseases which have an influence on nutritional status, including congenital heart disease, renal failure, sickle cell disease or liver disease and their mothers were also excluded from both study groups. Also excluded were children who had been in the nutritional rehabilitation programme for more than 7 days and their mothers. Children who were severely ill were also excluded until they were stable, if this was within the 7 days.

### Sampling

Purposive sampling was used in this study. We recruited consecutive patients with MAM and SAM admitted to the malnutrition ward or referred to the nutritional rehabilitation unit into the study between 9^th^ January and 10^th^ June 2013 who met weight-for height and other inclusion criteria, and gave consent. A comparative group of children attending PML who were being seen or treated for conditions other than malnutrition were recruited from the out-patients department and from the general paediatric wards if they had a weight-for-height z score of < −2SD, met inclusion criteria and gave consent. These were classified as controls but were not matched by age or sex to the cases.

We had some challenges recruiting controls especially from the general wards as many of those screened did not meet the criteria for being “well nourished”. Thus we extended the time of recruitment of the comparison group to 10^th^ September 2013 due to difficulty obtaining suitable controls and because of an industrial action which reduced patient attendance.

### Measurements and data collection

A Class III infant scale (Seca 334) was used to measure the children’s weight. A Seca 417 measuring board was used to measure length while height measurements were done using a Leicester height measure. These were recorded to the nearest millimetre. MUAC and head circumference were done using non-stretch tape measures. Research personnel making these measurements were trained in standardized techniques for performing these measurements. A Royal College of Paediatrics and Child Health training video clip was used as part of the training.

Weight-for-height measures wasting or acute malnutrition and can be expressed as a z-score which is the number of standard deviations or Z-scores below or above the reference mean or median value [21]. The Mid-Upper Arm Circumference (MUAC) is the arm circumference taken at the midpoint between the tip of the shoulder (acromium process) and the tip of the elbow (olecranon process). Both measurements measure wasting or acute malnutrition but correlation between them is often poor. MUAC is better predictor of mortality, easier and less cumbersome to perform and therefore is recommended for use in community-based screening [22].

A semi-structured questionnaire and a data record form were used to collect the information on the child’s profile. The information collected included data on the child’s age, sex, birth weight and birth order, maturity and problems at birth, child development, HIV status, chronic illness, illness episodes and diarrhoeal episodes over the past year. Information on nutritional status, sources of nutrition advice, growth pattern, immunisation status and preventive interventions such as de-worming, vitamin A supplementation and antenatal and postnatal visits was also obtained.

Information on faltering growth was obtained from the Child Health Record and in this study it was defined as a fall off the growth curve through two or more centile spaces on the growth chart. At the time, adequacy of antenatal visits was defined as 4 or more antenatal visits and postnatal visits as two or more postnatal visits.

### Statistical analysis

The data were entered into a Microsoft Access (Microsoft Corporation, Redmond, Washington) and analysed using Stata 11.0® (College Station, Texas 77845 USA). Classification of malnutrition using weight for length/height measurements was done using the WHO Anthro for personal computers, version 3.2.2, 2011. Frequencies and means were computed. The results were presented using tables, graphs with statistical inference. Both univariate and multivariate analysis were done to determine factors associated with malnutrition with the variables grouped under socio-economic and demographic factors, health outcomes and uptake of interventions. Variables significant at *p* < 0.2 in the univariate analysis were entered into the final multivariate analysis model. Statistical significance was accepted at a 5 % probability level, i.e. a *p*-value of less than 0.05.

### Ethics

Ethical approval was sought and obtained from the University of Ghana Medical School’s Ethical and Protocol Review Committee [Protocol Identification Number: MS-Et/M.8-P.5.8/2011-2012]. Ethical approval was also obtained from the Ghana Health Service Ethical Review Committee [Protocol Identification Number GHS-ERC 05/07/2012]. Written consent was obtained from the mothers/guardians of the children using consent forms which were signed or thumb printed.

## Results

### Description of the study participants

Table 1 shows the socio-economic and demographic description of the study participants. A total of 371 children participated in the study involving 182 malnourished children and 189 well-nourished children and their mothers. Female children constituted 52.7 % (*n* = 96) and 47.6 % (*n* = 90) of the malnourished and well-nourished groups respectively. More than half of the malnourished children were in the 6 months to 12 months age group with a median age of 11 months in the malnourished group. Or over 40 % of both groups were aged between 12 and 24 months. A total 86.0 % (*n* = 154) of mothers of malnourished children were educated and 93.5 % (*n* = 174) of mothers of well-nourished children were also educated. An assessment of the occupational status indicated that 18.1 % (*n* = 33) and 7.9 % (*n* = 15) of mothers of malnourished children and well-nourished children respectively were unemployed. Family income levels were >200 GH Cedis in 63.2 % (*n* = 115) and 87.8 % (*n* = 166) in malnourished and well-nourished children respectively.

**Table 1:** Socio-economic and demographic characteristics of 371 children and their mother's (caregivers) attending PML hospital in Accra, Ghana

Characteristic		Nutritional status of child	
		Malnourished n, %	Well-nourished n, %
Gender			
	Female	96 (52.7)	90 (47.6)
	Male	86 (47.3)	99 (52.4)
Age category			
	6-9 months	56 (30.8)	55 (29.1)
	10-11 month	38 (20.9)	27 (14.3)
	12-24 months	80 (44.0)	82 (43.4)
	25-59 months	8 (4.3)	25 (13.2)
Mother's educational status			
	Uneducated	25 (14.0)	12 (6.5)
	Educated	154 (86.0)	174 (93.5)
Mother's level of education (educated mothers)			
	Basic	112 (72.7)	93 (53.4)
	Post-basic	42 (27.3)	81 (46.6)
Mother's occupational status			
	Unemployed	33 (18.1)	15 (7.9)
	Employed	149 (81.9)	174 (92.1)
Monthly family income			
	≤200 GH Cedis^1^	67 (36.8)	23 (12.2)
	>200 GH Cedis^1^	115 (63.2)	166 (87.8)

^1^1.00$ = 2.00GH Cedis

Table 2 provides a description of the health outcomes of the study participants. A vast majority of the study participants recruited were out-patients comprising 72 % of the malnourished group and 90.5 % of the well-nourished group. There were four (4) cases of Kwashiorkor (oedematous SAM). Low birth weight was recorded in 13.9 % (*n* = 23) and 5.9 % (*n* = 10) of malnourished and well-nourished children respectively with developmental delay present in 15.9 % (*n* = 29) of malnourished children.

**Table 2:** Health outcomes of 371 children attending PML hospital in Accra, Ghana

Characteristic	Nutritional status of child	
	Malnourished n, %	Well-nourished n, %
Admission status		
In-patient	51 (28.0)	18 (9.5)
Out-patient	131 (72.0)	171 (90.5)
Birth weight		
Low (<2.5 kg)	23 (13.9)	10 (5.9)
Normal (≥2.5 kg)	142 (86.1)	159 (94.1)
An episode of diarrhoea (within last 6 months)		
Yes	122 (67.0)	76 (40.2)
No	60 (33.0)	113 (59.8)
Developmental delay		
Yes	29 (15.9)	3 (1.6)
No	153 (84.1)	186 (98.4)
Hospital admission (in the past one year)		
Yes	82 (45.1)	46 (24.3)
No	100 (54.9)	143 (75.7)
Passage of worm status (in last 6 months)		
Passed worm	6 (3.3)	3 (1.6)
No worm passed	176 (96.7)	186 (98.4)
Sickness episode (in past 1 month)		
Yes	110 (60.4)	104 (55.0)
No	72 (39.6)	85 (45.0)

Table 3 is a description of uptake of interventions of the study participants. Inadequate number of antenatal visits (20.9 %, *n* = 38) and postnatal visits of less than two (27.5 %, *n* = 50) were reported in mothers of malnourished children. Only 6.6 % (*n* = 12) of malnourished children were de-wormed in the last six months compared with 20.6 % (*n* = 39) of well-nourished children. Assessment of the child health record booklet indicated that faltering growth had occurred in 77.2 % (*n* = 71 and 19.5 % (*n* = 24) of malnourished and well-nourished children respectively (Table 3). The proportion of mothers who had received breastfeeding and nutritional counselling was high in both study groups (93.4 % in malnourished group and 99.5 % in well-nourished group) (Table 3). Whereas the delivery room was reported as the commonest setting for nutritional counselling by mothers of malnourished children (39.3 %, *n* = 57), the child health record book was reported as the commonest source by mothers of well-nourished children (35.4 %, *n* = 62). The uptake of BCG vaccines was high (99.4 %) in both the malnourished group and the well-nourished group. The pentavalent vaccine was taken by 97.1 % (*n* = 167) of the malnourished children and 98.2 % (*n* = 164) of the well-nourished children (Table 3).

**Table 3:** Uptake of interventions of 371 children and their mother's (caregivers) attending PML hospital in Accra, Ghana

Characteristic		Nutritional status of child	
		Malnourished n, %	Well-nourished n, %
Number of antenatal visits			
	Inadequate	38 (20.9)	10 (5.3)
	Adequate	144 (79.1)	179 (94.7)
Number of postnatal visits			
	< Two visits	50 (27.5)	24 (12.7)
	≥ Two visits	132 (72.5)	165 (87.3)
De-worming status (in last 6 months)			
	Not de-wormed	170 (93.4)	150 (79.4)
	De-wormed	12 (6.6)	39 (20.6)
Mother received breastfeeding and nutritional counselling			
	No	12 (6.6)	1 (0.5)
	Yes	170 (93.4)	188 (99.5)
Growth monitoring indicator status			
	Faltering	71 (77.2)	24 (19.5)
	Growing well	21 (22.8)	99 (80.5)
BCG vaccine			
	No	1 (0.6)	1 (0.6)
	Yes	171 (99.4)	166 (99.4)
Penta-3 vaccine			
	No	5 (2.9)	3 (1.8)
	Yes	167 (97.1)	164 (98.2)
Measles vaccine			
	No	22 (15.0)	14 (9.9)
	Yes	125 (85.0)	128 (90.1)
Vitamin A status			
	Not up to date	44 (25.6)	39 (23.4)
	Up to date	128 (74.4)	128 (76.6)

### Socio-economic and demographic factors associated with malnutrition

Table 4 shows the socio-economic and demographic factors associated with malnutrition in the study participants. Gender, mother's educational status and mother's occupational status were not associated with malnutrition in the multivariate analysis (*p* > 0.05) (Table 4). Children who were 24 months and below had higher odds of being malnourished compared with those of 25–59 months (Adjusted OR = 4.13 [95 % CI, 1.64-10.40], *p* = 0.003). Similarly, family income levels of ≤200 GH Cedis was associated with higher odds of malnutrition compared with income levels of >200 GH Cedis (Adjusted OR = 4.23 [95 % CI, 2.41-7.44], *p* < 0.001).

**Table 4:** Socio-economic and demographic factors associated with malnutrition in 371 children attending PML hospital in Accra, Ghana

Characteristic		Crude OR [95 % CI]	*p*-value	Adjusted OR [95 % CI]^2^	*p*-value
Gender					
	Female	1.23 [0.80-1.88]	0.323	-	-
	Male	1.00			
Age category					
	≤24 months	3.32 [1.40-8.73]	0.003	4.13 [1.64-10.40]	0.003
	25-59 months	1.00		1.00	
Mother’s educational status					
	Uneducated	2.35 [1.09-5.31]	0.017	2.06 [0.95-4.47]	0.067
	Educated	1.00		1.00	
Mother's occupational status					
	Unemployed	2.57 [1.29-5.28]	0.003	1.93 [0.97-3.84]	0.061
	Employed	1.00		1.00	
Monthly family income					
	≤200 GH Cedis^1^	4.20 [2.41-7.48]	<0.001	4.23 [2.41-7.44]	<0.001
	>200 GH Cedis^1^	1.00		1.00	

^1^1.00$ = 2.00GH Cedis; ^2^Varibales with *p* < 0.2 in the univariate analysis were entered into the multivariate analysis model; OR = Odds ratio; CI = Confidence interval

### Heath outcomes associated with malnutrition

Table 5 shows the health outcomes associated with the uptake of interventions. In the multivariate analysis, children who had low birth weight (Adjusted OR, 2.65 [95 % CI, 1.09-6.45], *p* = 0.032) or showed evidence of developmental delay (Adjusted OR, 12.09 [95 % CI, 2.68-54.57], *p* = 0.001) were associated with higher odds of malnutrition (Table 5). Similarly, children with an episode of diarrhoea (within the last 6 months) had a higher odds of malnutrition (Adjusted OR, 2.23 [95 % CI, 1.36-3.66], *p* = 0.002).

**Table 5:** Health outcomes associated with malnutrition in 371 children attending PML hospital in Accra, Ghana

Characteristic		Crude OR [95 % CI]	*p*-value	Adjusted OR [95 % CI]^2^	*p*-value
Age category					
	≤24 months	3.32 [1.40-8.73]	0.003	4.65 [1.73-12.55]	0.002
	25-59 months	1.00		1.00	
Monthly family income					
	≤200 GH Cedis^1^	4.20 [2.41-7.48]	<0.001	2.93 [1.59-5.42]	0.001
	>200 GH Cedis^1^	1.00		1.00	
Admission status					
	In-patient	3.70 [2.01-7.04]	<0.001	2.10 [0.98-4.50]	0.057
	Out-patient	1.00		1.00	
Birth weight					
	Low (<2.5 kg)	2.58 [1.13-6.26]	0.014	2.65 [1.09-6.45]	0.032
	Normal (≥2.5 kg)	1.00		1.00	
An episode of diarrhoea (within last 6 months)					
	Yes	3.02 [1.94-4.73]	<0.001	2.23 [1.36-3.66]	0.002
	No	1.00		1.00	
Developmental delay					
	Yes	11.75 [3.52-61.11]	<0.001	12.09 [2.68-54.57]	0.001
	No	1.00		1.00	
Hospital admission (in the past one year)					
	Yes	2.55 [1.60-4.07]	<0.001	1.70 [0.93-3.10]	0.086
	No	1.00		1.00	
Passage of worm status in last 6 months					
	Passed worm	2.11 [0.44-13.23]	0.285	-	-
	No worm passage	1.00			
Sickness episode (in past 1 month)					
	Yes	1.25 [0.81-1.93]	0.291	-	-
	No	1.00			

^1^1.00$ = 2.00GH Cedis; ^2^Varibales with *p* < 0.2 in the univariate analysis were entered into the multivariate analysis model in addition to age category and family income level; OR = Odds ratio; CI = Confidence interval

### Uptake of interventions to prevent malnutrition

Table 6 shows the uptake of reproductive health and child health interventions that prevent malnutrition including antenatal and postnatal care, de-worming, breastfeeding and nutrition counselling, growth monitoring and immunisation. In the multivariate analysis, the inadequate or lack of antenatal visits was marginally associated with malnutrition (Adjusted OR, 4.31 [95 % CI, 1.01-19.12], *p* = 0.049), whereas the number of postnatal visits was not (*p* > 0.05). The odds of being malnourished was 8.47 times higher in children who had not been de-wormed (in the last six months) compared with children had been de-wormed (Adjusted OR, 8.47 [95 % CI, 1.99-36.01], *p* = 0.004) (Table 6). Faltering growth recorded during growth monitoring was also associated with an increased odds of a child being malnourished (Adjusted OR, 21.40 [95 % CI, 8.74-52.41], *p* < 0.001) in the multivariate analysis (Table 6). There was no significant difference between the uptake of the three immunisation vaccines or Vitamin A by children who were malnourished and those who were well-nourished (*p* > 0.05) (Table 6).

**Table 6:** Uptake of interventions associated with malnutrition in 371 children attending PML hospital in Accra, Ghana

Characteristic		Crude OR [95 % CI]	*p*-value	Adjusted OR [95 % CI]^2^	*p*-value
Age category					
	≤24 months	3.32 [1.40-8.73]	0.003	1.90 [0.38-9.48]	0.431
	25-59 months	1.00		1.00	
Monthly family income					
	≤200 GH Cedis^1^	4.20 [2.41-7.48]	<0.001	1.98 [0.65-6.01]	0.229
	>200 GH Cedis^1^	1.00		1.00	
Number of antenatal visits					
	Inadequate/none	4.72 [2.21-10.96]	<0.001	4.31 [1.01-19.12]	0.049
	Adequate	1.00		1.00	
Number of postnatal visits					
	< Two visits	2.60 [1.48-4.67]	<0.001	0.84 [0.27-2.55]	0.753
	≥Two visits	1.00		1.00	
De-worming status (in last 6 months)					
	Not de-wormed	3.68 [1.80-8.00]	<0.001	8.47 [1.99-36.01]	0.004
	De-wormed	1.00		1.00	
Received breastfeeding/nutritional counselling					
	No	13.27 [1.92-570.13]	0.002	Not estimable^3^	Not estimable
	Yes	1.00			
Growth monitoring indicator status					
	Faltering	13.95 [6.87-28.56]	<0.001	21.40 [8.74-52.41]	<0.001
	Growing well	1.00		1.00	
BCG vaccine					
	No	0.97 [0.01-76.65]	0.983	-	-
	Yes	1.00			
Penta-3 vaccine					
	No	1.64 [0.31-10.69]	0.501	-	-
	Yes	1.00			
Measles vaccine					
	No	1.61 [0.75-3.56]	0.189	0.56 [0.15-2.02]	0.373
	Yes	1.00		1.00	
Vitamin A status					
	Not up to date	1.13 [0.67-1.91]	0.633	-	-
	Up to date	1.00			

^1^1.00$ = 2.00GH Cedis; ^2^Varibales with *p* < 0.2 in the univariate analysis were entered into the multivariate analysis model in addition to age category and family income level; ^3^Not estimable due to coll inearity; OR = Odds ratio; CI = Confidence interval

A total of 80 of the children have had one episode of diarrhoea comprising 26.4 % of malnourished children and 16.9 % of well-nourished children. Two or more episodes of diarrhoea were reported by 40.7 % of the cases and 23.3 % of the controls. Eleven malnourished children (6.0 %) had 4 or more episodes of diarrhoea compared with 2.1 % (*n* = 4) of the well-nourished children.

## Discussion

### Socio-economic and demographic factors

In this study more than half of the malnourished children were in the 6 months to 12 months age group (Table 1). Since this coincides with the weaning period, it may well be that inappropriate weaning or complementary feeding practices may have been a major contributor to this finding [3, 14]. A similar pattern was found in a study of admissions of children under the age of five years with protein energy malnutrition in Enugu, Nigeria [23]. The study on malnutrition at PML in the 1990’s differs in methodology from our study as the researchers specifically targeted children between 8 and 36 months. The average age then was around 14 months for underweight and 17 months for severe malnutrition [6].

We found that an age of 24 months or less was associated with malnutrition in the multivariate analysis. It is well known that this age group is most vulnerable to malnutrition and its effects [24]. At the same time the age group provides a window of opportunity for intervening to reduce the effects of malnutrition hence the emergence of the Scaling Up Nutrition (SUN) movement which aims at mitigating nutritional problems during pregnancy, and in this age group [24, 25]. It is a country-led process which brings organizations together to support nations to implement nutrition interventions in their national plans through multidisciplinary working.

A monthly family income of ≤200 GH Cedis (≤100 USD) was associated with malnutrition in the multivariate analysis reiterating the importance of poverty in the aetiology of malnutrition in this setting [26]. This is similar to a previous study in Ghana which found that economic inequality is strongly associated with chronic under - nutrition. It is also similar to a study in Nigeria which found that maternal monthly income < $20, monthly household food expenditure of < $55 was associated with malnutrition [26]. In contrast, the educational status of mothers and their occupational status in this study were only significantly associated with malnutrition in the univariate analysis and not in the multivariate analysis. The researchers in Nigeria also found that malnutrition was significantly. associated with education below secondary level in a univariate but not in multivariate analysis of its determinants [27]. They also found that residence in a one room apartment, higher birth order and incomplete immunization of the child were significantly associated with malnutrition in that study consequently, they suggested a multidisciplinary approach for preventive strategies just as the SUN movement has done [27]. We did not find an association between immunisation status and malnutrition possibly because immunisation rates were similar in both groups and was high. It could be that making a health service such as immunisations readily accessible reduces the effects of poverty and health inequalities.

Although poverty can exert its influence on all three arms of the UNICEF conceptual frame work of underlying causes of malnutrition, it has a major effect on household food security [28]. Food security is determined by several factors including food prices, agricultural practices, climate change and market forces among others [29]. There was a gradual increase in the number of people worldwide who were underweight from 1990, peaking in 2008. This was worsened by the global recession in 2008 and 2009 which particularly affected the urban poor. It led to price hikes in food, limiting food consumption and causing a shift to less balanced diets. It also left less resources for buying goods and services to ensure hygienic practices, health and well being [29]. Suggestions have been made to counteract this by promoting agricultural growth, measures to reduce extreme market volatility, and expansion of social protection and child nutrition action particularly nutrition sensitive interventions [29–31].

In response to this call, there have been several studies exploring the use of social protection measures such as cash transfers to mitigate the effects of poverty on malnutrition in childhood and some have been particularly successful as reported in a study in Niger [32]. This study found that preventive distributions of supplementary food and cash transfer were better at preventing MAM and SAM than either of these measures alone. However, it is not clear how this can be sustained in the long term. In any case it is rewarding to note that there are plans to ensure that strong social protection measures are enshrined in the upcoming sustainable development goals.

### Health outcomes

The results of the study showed that low birth weight, having an episode of diarrhoea within the last 6 months and the presence of developmental delay were all associated with malnutrition in the multivariate analysis. Similar findings have been reported by other studies [3, 6, 23]. On the other hand, although having been admitted to hospital within the past one year was associated with malnutrition in the univariate analysis, the association was not statistically significant in the multivariate analysis. There was also no relationship between sickness episodes and malnutrition unlike the study by Maleta et al. [33] in Malawi which found that malnutrition was associated with frequent illness episodes in infancy. It is possible that we may have found an association if we had focussed on infancy as they did.

Diarrhoeal diseases are generally more frequent and tend to be more severe in malnourished children because of the association between malnutrition and infection [1–3]. In this study, 40.7 % of the malnourished children had two or more episodes of diarrhoea compared to 23.3 % of the controls. This suggests that efforts to control diarrhoea are important for effective prevention of malnutrition. This must include providing effective advice on feeding during diarrhoea episodes and adequate follow up after each episode. It is also important that protocols exist for investigating and managing children who have relapsed after previous treatment for diarrhoea to exclude underlying medical conditions [23]. Developmental delay was more prevalent in malnourished children and associated with increased odds of being malnourished. Malnutrition often causes developmental delay however malnutrition can also be precipitated in feeding difficulties due to a chronic neurological problem [9]. It appears this association was more of an effect rather than a cause of malnutrition in most of the children. However eight (8) out of the 29 malnourished children with developmental delay were reported to have had problems at birth and one (1) had a chronic neurological condition, cerebral palsy which could have precipitated the delay in data not presented here. Early intervention will ensure that these children make the most of their developmental potential to reduce the effect of malnutrition [30, 34, 35].

### Uptake of interventions

The study found that inadequate/lack of antenatal care was associated with malnutrition in the multivariate analysis although the association was marginal. This implies that mothers of malnourished children were less likely to have had adequate health contacts through antenatal visits. The present result is similar to a study in three Latin American countries which found only a weak association between antenatal care and reduction in the level of child malnutrition and some variations between countries [10]. They attributed these findings to differences in the quality of care and health inequalities.

Antenatal care provides opportunities for nutritional counselling which mothers of well nourished children may have benefited from and it has been shown to be effective if there is food security [11, 13, 14]. It is also worthy to note that the mothers of malnourished children reported the delivery room to be the main setting for nutritional counselling, whereas mothers of well nourished children reported the child health record books as their main source of nutritional advice. The study also shows that mothers of well nourished children were more likely to de-worm their children every 6 months. Regular de-worming of children has been reported to be a useful intervention for preventing malnutrition in some settings and this appears to be one [11, 14]. Furthermore, a majority of the mothers reported that they had nutritional counselling or advise from the health service which is one of the interventions expected in a national plan [16, 17, 24]. The delivery room is an important setting for counselling mothers on early initiation of breast feeding [36].

Since maternity care is free in all government health institutions, pregnant women should be encouraged to access antenatal care and the health serivice should engage those mothers who miss out through home visiting. However a more specific and targeted approach will be needed. Vitamin A supplementation was not associated with malnutrition even in the univariate analysis. Although Vitamin A reduces child mortality, it is not known to affect anthropometric measurements [14].

Growth faltering occurred in both groups; however it was significantly more common in children with malnutrition and was still significant after multivariate analysis. This is not an unexpected finding. We encountered some incomplete records which are most likely because most mothers come for growth monitoring only when their child’s immunisations are due. They used to stop at 9 months after the measles immunisation but more recently this has gone up to 18 months since the second dose of measles was introduced. Additional clinic visits may be necessary to pick up and monitor children who are faltering between the ages of 9 and 18 months and above. The usefulness of these visits needs to be established first since growth monitoring has been used in several intervention programmes with mixed results [11, 14].

The main limitation of this study was a challenge related to the classification of malnutrition. Weight for height criteria was used to make the results comparable to other studies. The WHO recommends the use of both MUAC and weight for height as independent criteria for classifying malnutrition whereas nutrition rehabilitation centres in Ghana including PML use only MUAC [20]. We found that about a third of patients who would have passed as being well-nourished using MUAC criteria could not be included in the control group because they were malnourished using weight-for-height criteria. This means that there may be several malnourished children who are missed each day. Missed opportunities for picking up malnourished children has been reported in several studies, including a study in a teaching hospital in Ghana [37–39]. Understandably, MUAC is an easier and more practical measurement in small peripheral health facilities. However, in larger health facilities like PML and teaching hospitals, it should be possible to do weight and height measurements routinely and hence record weight for height measurements. Further studies are needed to assess the effect of using either criteria on the prevalence and cost-benefit of management as it may well be that the burden of malnutrition in the hospital is much higher than we are treating.

Children with Kwashiorkor who could not stand to have their heights measured or were too ill were not included. There was a slight over representation of older children among the well nourished children. Also the patients were not matched so it is possible that this may have created a bias. We also recognise that children labelled as well-nourished are likely to contain some children with mild malnutrition; however, for the purposes of this study, we have classified them as controls in line with current classification of malnutrition.

## Conclusions

Malnutrition was associated with a monthly family income of ≤200 GH Cedis (≤100 USD) but not with maternal educational status and employment status which highlights a need to address poverty. Malnutrition was also associated with lack or inadequate antenatal care, not de-worming children regularly, low birth weight, previous diarrhoea episodes, and developmental delay. Though the latter three conditions could be consequences of malnutrition they could aggravate malnutrition through lack of health services. Thus preventing these conditions and providing adequate follow up for diarrhoea patients will be important steps in preventing malnutrition in this population. Interventions to reduce malnutrition were generally better patronised by the mothers of well nourished children. Efforts must be made to reach mothers who default on antenatal visits and de-worming their children regularly. Furthermore, growth monitoring should be encouraged in this setting and further studies on the timing and use of information from the activity are needed.

## Abbreviations

MAM: Moderate acute malnutrition
MUAC: Mid - upper arm circumference
SAM: Severe acute malnutrition
PML: Princess Marie Louise Children’s Hospital
